# Does MYO and ALA Supplementation Improve PCOS Outcomes?

**DOI:** 10.3390/medicina61050885

**Published:** 2025-05-13

**Authors:** Selma Firat, Koray Elter, Sinan Ateş, Mehmet Fisunoğlu

**Affiliations:** 1 Department of Nutrition and Dietetics, Faculty of Health Sciences, Kırklareli University, Kırklareli 39100, Turkey; 2 Department of Obstetrics and Gynecology, Faculty of Medicine, Trakya University, Edirne 22030, Turkey or korayelter@hotmail.com (K.E.); sinanates@trakya.edu.tr (S.A.); 3 Department of Nutrition and Dietetics, Faculty of Health Sciences, Hacettepe University, Ankara 06100, Turkey; fisunogl@hacettepe.edu.tr

**Keywords:** PCOS, myoinositol, lipoic acid, insulin resistance, dietary supplementation

## Abstract

*Background and Objectives*: This study aimed to evaluate the impact of myoinositol (MYO) and α-lipoic acid (ALA) supplementation on hormonal and metabolic markers in women diagnosed with polycystic ovary syndrome (PCOS). *Materials and Methods*: A retrospective case–control study was conducted with 58 women aged between 18–40 years who met the Rotterdam criteria for PCOS. The case group (*n* = 29) received MYO (2000 mg/day) and ALA (400 mg/day) supplements, while the control group (*n* = 29) did not receive any treatment. Data on the subjects’ anthropometric measures, glycemic indices, sex hormones, and lipid profiles were collected. *Results*: The results demonstrated that, following three months of MYO + ALA supplementation, the case group exhibited steady body weight (*p* = 0.484) and BMI (*p* = 0.405), whereas the control group demonstrated a significant increase in both (*p* = 0.029; *p* = 0.026, respectively). A stratified analysis based on BMI, waist circumference, and waist-to-height ratio revealed that HbA1c (%) was significantly lower in the “normal” subgroup compared to the “risky” subgroup within the case group (*p* < 0.05). Although the mean HbA1c, insulin, and HOMA-IR values were comparable between the two groups, the LH/FSH ratio significantly increased in the control group (*p* = 0.010). No significant differences were observed in the lipid profiles between the two groups; however, LDL levels decreased significantly in the case group (*p* = 0.024). Across all classifications, the “normal” subgroup consistently exhibited lower HbA1c and TG/HDL ratios than the “risky” subgroup. *Conclusions*: Adding MYO + ALA supplementation to standard PCOS treatment may offer metabolic benefits, particularly in maintaining glycemic control, body weight, and BMI. Supplementation also reduces LDL.

## 1. Introduction

Polycystic ovary syndrome (PCOS) is a common condition in women, affecting both reproductive and overall health [1]. One of its symptoms, hirsutism, is characterized by elevated ovarian and adrenal androgen secretion, which often leads to hyperandrogenic signs such as acne, alopecia, menstrual irregularities, anovulation, and infertility [2]. The prevalence of polycystic ovary syndrome (PCOS) varies significantly, ranging from 6% to 20% among women of reproductive age. This variation is primarily attributed to the use of different diagnostic criteria, including the Rotterdam criteria, the NIH criteria, and the AE-PCOS Society guidelines, each emphasizing distinct clinical or biochemical features. Additionally, differences in study populations, such as ethnicity, age distribution, and lifestyle factors, further contribute to the variability in reported prevalence [3,4,5]. The exact cause of PCOS is still unknown; however, existing literature suggests it may be the result of a complex interaction between genetic predisposition, prenatal androgen exposure, epigenetic changes, and environmental factors [6,7]. Beyond endocrine dysfunction, persistent insulin resistance (IR), hyperinsulinemia, obesity, and metabolic syndrome (MetS) form the underlying pathophysiological basis for associated health risks, including type 2 diabetes, cardiovascular disease, hypertension, atherosclerosis, and dyslipidemia. Hyperinsulinemia and insulin resistance (IR) contribute to increased ovarian androgen production and a reduction in serum sex hormone-binding globulin (SHBG) levels, leading to higher circulating concentrations of free testosterone. This interplay between IR and ovarian hyperandrogenism highlights the direct role of insulin in modulating ovarian function [8]. Although there is no cure for PCOS, treatments can alleviate symptoms. Insulin-sensitizing agents are commonly used in women with PCOS due to the frequent presence of IR and hyperinsulinemia. Typical treatment modalities used in women with PCOS include lifestyle changes and drug therapy such as clomiphene citrate, aromatase inhibitors, low doses of human menopausal gonadotropin or FSH, insulin sensitizers, laparoscopic ovarian drilling, and in vitro fertilization (IVF) [9]. Various therapeutic agents with insulin-sensitizing properties are employed to address these metabolic and hormonal disturbances in PCOS [10]. Among these, inositols and alpha-lipoic acid (ALA) have gained attention; inositols act as insulin sensitizers, while ALA offers additional antioxidant effects [10]. Both supplements have demonstrated the potential to improve insulin sensitivity, along with hormonal and metabolic parameters in women with PCOS. When ALA is given alone or in combination with MYO to women with PCOS, an improvement in their clinical and metabolic features has been observed [11,12,13].

Inositol (myoinositol and di-chiroinositol) is a dietary supplement involved in insulin signal transduction as a secondary messenger and shown to be effective in the treatment of PCOS [14]. Inositol is involved in the post-receptor signaling of several hormones such as insulin, follicle-stimulating hormone (FSH), and thyroid-stimulating hormone (TSH). MYO and DCI play a crucial role in the treatment of PCOS by improving metabolic regulation. MYO enhances insulin sensitivity, helping to restore ovulation and balance hormones in women with PCOS. Recent studies have shown that MYO is effective in reducing insulin resistance and regulating ovulation. However, its effects are limited in treatment-resistant cases, highlighting the need for further research [15,16].

MYO is one of the most widely used isoforms of inositol [17,18]. MYO can be incorporated into inositol phosphoglycan (IPG), a membrane phospholipid involved in insulin signal transduction. The interaction of its receptor with insulin can activate this transduction pathway mediated by inositols and enable the formation of intracellular messengers that allow the incorporation of glucose into oxidative metabolism instead of non-oxidative metabolism. MYO-IPG may reduce IR and improve glucose metabolism [17]. Systematic reviews have reported that ovulation rate and menstrual cycles in women with PCOS may improve with inositol supplementation [14,19].

Recently, the use of α-lipoic acid (ALA) has also been recognized as a possible therapeutic approach for the treatment of PCOS and IR [20]. ALA and its reduced form, dihydro-lipoic acid (DHLA), is a potent antioxidant that neutralizes reactive oxygen species (ROS) and can regenerate other antioxidant molecules [21]. ALA may improve insulin sensitivity by activating AMPK, a cellular energy sensor that induces translocation of GLUT4 to the plasma membrane via an insulin-independent mechanism in metabolism [22,23,24]. The effect of ALA on individuals with PCOS can be attributed to several mechanisms. Firstly, it acts as an antioxidant. Secondly, it exerts an inhibitory effect on the inflammatory pathway mediated by NF-κB, preventing its translocation to the nucleus and thereby playing an anti-inflammatory and immunomodulatory role by reducing proinflammatory cytokine release. Thirdly, ALA may improve insulin sensitivity and reproductive function and rectify metabolic abnormalities by increasing glucose uptake and playing a remarkable role in the insulin metabolic pathway [25]. ALA has been studied for its potential benefits in improving insulin sensitivity and metabolic function in women with PCOS. While ALA does not significantly affect reproductive hormones, it has shown promise in reducing insulin resistance and improving glucose metabolism. Combining ALA with other treatments, such as MYO, may enhance its effectiveness in managing metabolic symptoms in PCOS patients. However, further research is needed to fully understand the synergistic effects of this combination [26].

Many PCOS patients experience compensatory hyperinsulinemia, which can occur independently of overweight or obesity, indicating a natural predisposition of PCOS to this metabolic dysfunction [27]. BMI is a strong predictor of metabolic changes in women with PCOS at any age, and obesity is linked to the development of metabolic complications [28]. Based on such evidence, the objective of this study was to examine the effects of MYO and ALA supplementation on hormonal and metabolic markers in patients diagnosed with normal/overweight PCOS.

## 2. Material and Method

### 2.1. Subjects

This study was conducted between June 2021 and June 2022 with 58 patients, aged between 18 and 40 years in the Infertility Outpatient Clinic of the Department of Obstetrics and Gynecology at Trakya University Hospital. The study population was recently diagnosed with PCOS according to the Rotterdam Criteria. Written consent was obtained from all participating patients.

The diagnosis of PCOS was established according to the Rotterdam Criteria [1], with the presence of at least two criteria: (i) menstrual irregularities with intervals exceeding 45 days, (ii) clinical manifestations (such as acne and hirsutism) or biochemical indicators of hyperandrogenism, and (iii) detection of micro polycystic ovaries during ultrasound examination. Patients with any genetic or chronic diseases were excluded.

The sample size calculation was based on the study by Cianci et al. [29] To detect a significant difference in HOMA-IR values at Cohen’s d = 0.73 level, the minimum required sample size for the case group was determined to be 27 (α = 0.05, 95% confidence interval, 1 − β = 0.80). This calculation was made to ensure sufficient statistical power for the study, aiming to generate reliable results.

**Figure 1:**
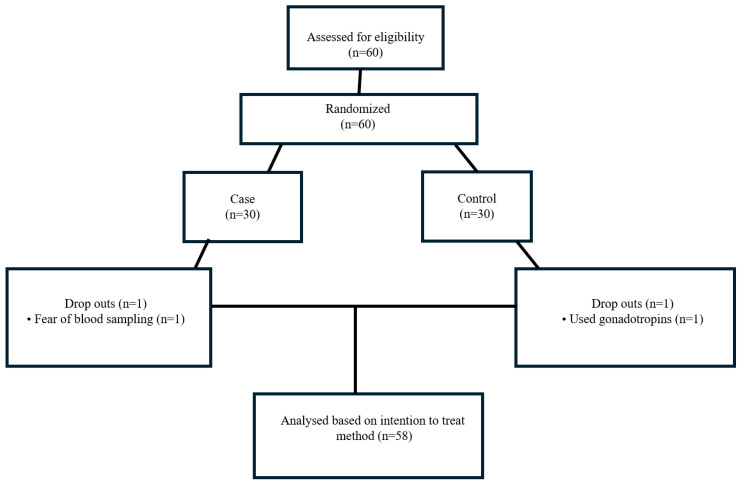
Study flow diagram.

This case–control retrospective study included 60 patients diagnosed with PCOS who were divided into two groups based on supplement use. However, 2 patients were excluded from the study because 1 of them did not want to give blood and 1 of them was using gonadotropin (Figure 1). The case group consisted of 29 patients who had used MYO (2000 mg/day) and ALA (400 mg/day) supplements for at least three months, without receiving any pharmacological treatment for PCOS. The control group included 29 patients with PCOS who did not use these supplements. Neither the case nor the control group was undergoing medication therapy, but all were under the supervision of physicians. All participants were of Turkish ethnicity. To control for potential confounding effects of lifestyle changes, participants were asked about any notable alterations in their routine behaviors—particularly physical activity—during the study period. Only those who reported no significant changes were included.

All participants were enrolled in a standard follow-up program for PCOS management. At baseline, the following assessments were conducted: clinical evaluation, ultrasound examination, hormonal and lipid profile measurements, and anthropometric evaluations, including weight. After 12 weeks of follow-up, the assessments were repeated, and additional parameters were collected, including three-day dietary records and an evaluation of hirsutism.

Patients in the case group were retrospectively interviewed at the end of the study regarding their adherence to the supplement regimen. Based on the collected data, all participants adhered to their respective treatment protocols without reporting adverse effects or discontinuing the study.

### 2.2. 3 Days Dietary Records and Assessment of Hirsutism

Each participant submitted detailed 3-day dietary records to ensure consistency in their normal dietary habits throughout the intervention period. These records were collected at the 12th week of the intervention. After determining the amounts of foods and beverages in grams, the amount of energy, macro, and micronutrients consumed was determined using the Nutrition Information Systems Package Program (BEBIS), a computer program [30]. The amounts of macro- and micronutrients determined for the calculation of the inflammatory burden gained from food were calculated according to Shivappa et al. [31]. The values were used to obtain dietary inflammatory index (DII) scores representing the inflammatory burden of the individual’s daily diet. A high dietary inflammatory index score was considered to be proinflammatory and a low dietary inflammatory index score was considered to be anti-inflammatory [32].

The Ferriman–Gallwey scoring method, a standard method for the objective evaluation of hirsutism, was used [33].

### 2.3. Biochemical Measurements

Hormonal evaluations were conducted at baseline on days 3–6 of the menstrual cycle. Lipid profile assessments included measurements of total cholesterol, high-density lipoprotein (HDL), low-density lipoprotein (LDL), and triglycerides (TG). Post-treatment endocrine assessments were conducted at least during the 12th week of treatment or a few days later, while still on treatment, to coincide with days 3–6 of the first menstrual cycle occurring after the 12-week treatment period. Insulin resistance was calculated using the Homeostasis Model Assessment–Insulin Resistance Index (HOMA-IR) formula. This value is obtained by multiplying the fasting glucose value by the fasting insulin and dividing the result by 405. The cut-off value for HOMA-IR was accepted as 2.5 [34]. TG/HDL and LH/FSH ratios were calculated.

### 2.4. Anthropometric Measurements

Body Mass Index (BMI) [35], body fat percentage (%) [36], muscle mass (kg), body water percentage [37], and basal metabolic rate (BMR) [38] measurements of the individuals participating in the study were calculated by using anthropometric formulas from body weight (kg) [39]. Waist circumference (WC) and hip circumference were measured. Waist and hip circumferences were measured using a non-elastic measuring tape with the participants standing upright. All measurements were performed by the same trained investigator following a standardized protocol. BMI values were calculated from weight and height measurements using the formula: BMI [weight (kg)/height (m)^2^]. The BMI classification of the World Health Organization (WHO) was used (<25 kg/m^2^ = underweight/normal, ≥25 kg/m^2^ = overweight/obese) [35]. The obtained WC measurements were evaluated according to the WHO classification [35]. A waist/hip ratio above 0.85 was classified as ‘risky’ and below 0.85 was classified as ‘no risk’ for women in the 2011 WHO Report [40]. Waist/height ratio: according to Ashwell’s classification; <0.4 ‘attention’, 0.4–0.5 ‘normal’, ≥0.5 ‘precautions should be taken’, 0.6 was classified as ‘high risk’ [41].

This study obtained approval from the T.C. Trakya University Medical Faculty Scientific Research Ethics Committee and was conducted according to the ethical guidelines of the 1975 Declaration of Helsinki. This study did not receive any funding support. This study complied with The Strengthening the Reporting of Observational Studies in Epidemiology (STROBE) statement.

### 2.5. Statistical Analysis

The statistical analyses were conducted using the software package SPSS (IBM SPSS Statistics 27). In the interpretation of the findings, frequency tables and descriptive statistics were employed. Parametric methods were used for variables that adhered to normal distribution. Accordingly, for the comparison of variables between two independent groups in line with parametric methods, the “Independent Sample-t” test (t-table value) was utilized, and for the comparison of two dependent groups, the “Paired Sample” test (t-table value) method was employed. Non-parametric methods were used for variables that did not conform to normal distribution. In line with non-parametric methods, the “Mann–Whitney U” test (Z-table value) was applied for the comparison of variables between two independent groups, and the “Wilcoxon” test (Z-table value) was used for the comparison of two dependent groups. When multiple comparisons were conducted, Bonferroni correction was applied to adjust the significance level. The significance of the treatment effects was reported as the mean differences along with their corresponding 95% confidence intervals. Statistical significance was defined as *p*-values < 0.05.

## 3. Results

**Figure 2:**
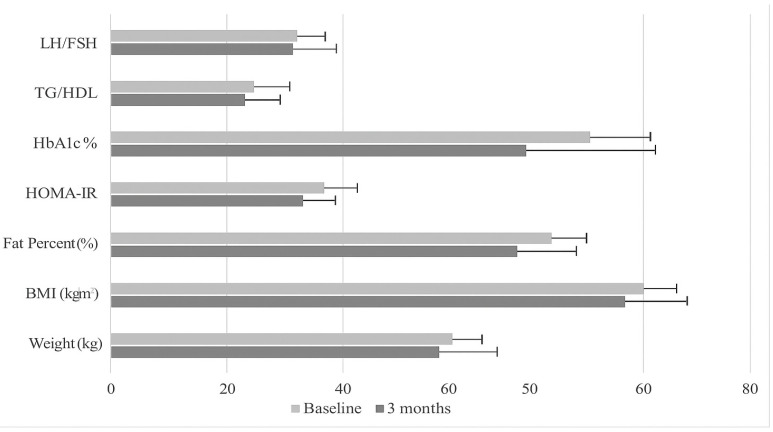
Effect of the association of ALA and MYO on hormone and PCOS-related metabolic markets (case group).

In the survey conducted, menstrual irregularity was assessed; the proportion of individuals with menstrual irregularity in the case group (72.4%) was found to be higher compared to the control group (41.4%). This difference is statistically significant (χ^2^ = 4.499, *p* = 0.034). Table 1 shows the patients’ mean age, anthropometric measurements, and body composition. The mean age of the groups was similar. There was no statistically significant difference in weight, BMI, fat percentage, fat mass, and fat-free mass in the case group (*p* > 0.05) (Table 1 and Figure 2). In the control group, a statistically significant increase in weight, BMI, and fat percentage compared to baseline was observed (*p* < 0.05). There was no statistically significant difference between the groups in terms of anthropometric measurements and body composition (*p* > 0.05).

Based on the 3-day dietary records collected during the intervention, no statistically significant differences were observed in the intake of dietary macro/micro-nutrients and DII scores between the groups. Patients with anti-inflammatory diets had higher energy and nutrient intake (*p* < 0.05).

Table 2 shows the relationships between the classifications based on anthropometric and biochemical measurements. In all classifications based on BMI, WC, waist/height, and waist/hip, the mean values of HbA1c and TG/HDL were significantly lower in patients classified as “normal” than in the ‘risky’ group (*p* < 0.05) (Table 2).

According to Table 3, the mean values of HbA1c (%), insulin (μU/mL), and HOMA-IR were not statistically significant in both groups, but the mean values were lower in the case group than baseline, whereas the opposite was the case in the control group (Figure 2). There has been no statistically significant change in the lipid profiles of both groups. LDL levels have significantly decreased in the case group (*p* = 0.024).

In general, no changes have been detected in the hormonal profiles of both groups. There was a statistically significant decrease in progesterone in both groups (*p* < 0.05). In the control group, the LH/FSH ratio has increased significantly (*p* = 0.010), while in the case group, it remains similar (*p* = 0.325).

In both groups, the mean CRP (mg/L) values increased significantly (case group: *p* = 0.041; control group: *p* = 0.048). In the intergroup evaluations of the subsequent biochemical parameters of the patients included in the study, it was found that the mean E2 (pg/mL) hormone values were significantly higher in the case group than in the control group (*p* = 0.015). No statistically significant difference was found between them in the analyses performed for other parameters (*p* > 0.05).

A positive, weak, and statistically significant correlation was found between the Ferriman–Gallwey score and androstenedione (ng/mL) (r = 0.262; *p* = 0.047). As androstenedione (ng/mL) increases, the Ferriman–Gallwey score will increase (Table 4).

## 4. Discussion

PCOS is a common female health problem characterized by disorders in the reproductive system and hormone balance. This study examined the effects of MYO + ALA supplements in patients with PCOS.

The results of this study showed that the proportion of individuals with menstrual irregularity was higher in the case group (72.4%) compared to the control group (41.4%), with a statistically significant difference (χ^2^ = 4.499, *p* = 0.034). These findings suggest that supplementation with MYO and ALA may influence menstrual regularity. However, while the data indicate a potential impact, the exact effect of these supplements on menstrual cycle normalization remains unclear. Further studies with larger sample sizes and more comprehensive evaluations are necessary to fully understand the long-term effects of MYO and ALA supplementation on menstrual health and regularity.

An analysis of the pre- and post-supplementation measurements in the case group revealed no statistically significant differences in body weight, BMI, fat percentage, or fat-free mass. However, a trend toward reductions in body weight, BMI, and fat percentage was observed following supplementation. This suggests that MYO + ALA supplements may be associated with weight management and body composition of patients with PCOS. In contrast to the case group, the control group showed a statistically significant increase in weight, BMI, and fat percentage from baseline. These results are consistent with the existing literature. Studies investigating the effects of MYO + ALA supplementation have reported reductions in BMI among supplement users. While some studies have found this decrease to be statistically significant [42,43,44], others have not observed a significant difference [11,27]. Fruzzetti et al. evaluated the duration-dependent effects of MYO (2000 mg) + ALA (800 mg) supplementation and found a statistically significant decrease in BMI at the end of the 6th month. However, measurements taken in the 12 and 24 months indicated an increase in BMI in the subsequent months [45]. A recent study examined the dose-dependent effects of MYO + ALA supplementation. The study included a total of 71 patients diagnosed with PCOS. The subjects received a daily dosage of 800 mg of ALA, with 43 patients also receiving 2 g of MYO and 28 receiving 1 g. The results demonstrated that the group receiving 2 g of MYO exhibited a substantial reduction in BMI, whereas the group receiving 1 g of MYO experienced a significant increase [46].

Insulin resistance is considered a key mechanism in PCOS, stemming from either ovarian steroidogenesis abnormalities or metabolic disturbances. Biochemical analyses showed that the mean HbA1c (%), insulin (μU/mL), and HOMA-IR values decreased in the case group after supplementation, although the changes were not statistically significant. In contrast, the control group exhibited higher mean values compared to the baseline. In our study, we observed modest improvements in glycemic control, as evidenced by changes in HbA1c levels. Although the decrease in HbA1c was not statistically significant, it suggests that MYO + ALA supplementation may have some potential in maintaining glycemic stability in women with PCOS. However, further large-scale studies are needed to confirm this hypothesis. These results are consistent with the findings in the literature. While some studies have reported a significant reduction in HOMA-IR and insulin levels following MYO + ALA supplementation [44,45,47,48], others have observed non-significant decreases [11,46]. MYO, integrated into IPG, aids insulin signaling by activating pathways that promote glucose utilization in oxidative metabolism, potentially reducing IR and improving glucose metabolism [17]. ALA enhances insulin sensitivity by activating AMPK, facilitating GLUT4 translocation independently of insulin. In PCOS, ALA acts as an antioxidant, inhibits NF-κB-mediated inflammation, and improves glucose uptake, insulin sensitivity, reproductive function, and metabolic balance [25]. Thus, it can be concluded that MYO + ALA supplementation may contribute to both metabolic and hormonal improvements in women with PCOS, particularly by assisting in weight management and supporting the maintenance of body composition. These improvements could have significant implications for managing the metabolic and reproductive symptoms of PCOS, although further research with larger sample sizes and long-term follow-up is needed to confirm these effects.

PCOS is like other chronic non-communicable diseases such as atherosclerosis, obesity, type 2 diabetes, and cardiovascular diseases, which are associated with low levels of inflammation [49,50]. Despite evidence linking inflammation and PCOS, uncertainty remains about the role of diet in controlling inter-individual variability in insulin resistance, hyperandrogenism, and chronic low-grade inflammation in PCOS. DII refers to the inflammatory index of the diet, a person’s diet may be generally healthy but at high risk of inflammation. Furthermore, medical nutrition therapy for PCOS patients can reduce hyperinsulinemia, hyperandrogenism, and inflammation, which may help control and reduce disease complications such as infertility [51]. In a study on postmenopausal women with metabolic syndrome, a six-month treatment with inositol and ALA combined with a low-calorie diet showed a statistically significant effect on HOMA-IR, both between baseline and the six-month follow-up and when compared to the placebo group. Additionally, a reduction in serum insulin levels was observed in 89.3% of patients taking the supplement [44].

Alongside glycemic control, we also assessed lipid profiles to determine if MYO + ALA supplementation has a broader impact on metabolic health. Analysis of the lipid profiles revealed a statistically significant decrease in mean LDL levels (mg/dL) in the case group (*p* < 0.05). These findings suggest that MYO + ALA may influence lipid metabolism, though the effect is modest and warrants further investigation. While LDL reduction may contribute to improved cardiovascular health, the modest change observed in this study warrants caution in interpretation. Further studies with a focus on long-term cardiovascular outcomes are needed. The TG/HDL ratio, a novel atherogenic index linked to insulin resistance and cardiovascular risk assessment, decreased in the case group but increased in the control group, though the changes were not statistically significant (*p* > 0.05). These findings are consistent with previous studies, which have reported non-significant improvements in certain lipid parameters [11,43].

Hormonal changes were similar between the groups. Both groups exhibited a statistically significant decrease in progesterone levels. In a study conducted on adolescents with PCOS, a significant reduction in 17-hydroxyprogesterone was reported, while LH, 17β-estradiol, delta-4-androstenedione, and testosterone levels remained unchanged [47]. Similarly, De Cicco et al. observed significant reductions in androstenedione and DHEAS levels following supplementation, whereas LH, FSH, E2, and prolactin levels showed non-significant decreases [11]. The present study demonstrated similar trends in hormonal parameters. Although statistically not significant, the mean LH/FSH ratio decreased in the case group, while a statistically significant increase was observed in the control group (*p* = 0.010). Consistent with these findings, Genazzani et al. reported a significant decrease in LH serum levels and the LH/FSH ratio following supplementation [27]. Additionally, another study found increases in FSH and TSH levels, alongside non-significant reductions in LH, E2, and prolactin levels [43]. The observed increase in E2 (pg/mL) hormone levels (*p* = 0.015) is statistically significant, but the biological mechanism underlying this change remains unclear. It is possible that MYO and ALA supplementation may influence insulin sensitivity and ovarian function, which could, in turn, affect estradiol secretion. However, further studies are needed to elucidate the specific pathways involved. Similarly, while no major changes were observed in other hormonal markers, small fluctuations were noted, particularly in the LH/FSH ratio. These changes, although not statistically significant, highlight the need for more comprehensive trials to explore the full hormonal impact of MYO + ALA supplementation. The variability of the sample numbers of the studies, the use of MYO + ALA supplements in different amounts and durations, and heterogeneous evaluation methods may have been effective on the significance level of the results.

Correlation analysis revealed a positive relationship between the Ferriman–Gallwey score and androstenedione. This suggests that hirsutism is related to androgen hormones.

Finally, regarding the symptoms of hirsutism, we observed a minor improvement in the Ferriman–Gallwey score. Although the clinical significance of this change is uncertain, it does suggest that MYO + ALA may have some effect on hyperandrogenic symptoms in women with PCOS. Further studies are necessary to better understand this relationship.

Although previous studies have also investigated the effects of MYO and ALA supplementation in women with PCOS, our findings contribute incremental evidence by examining a broader set of metabolic, hormonal, and inflammatory markers in a specific population. While the present study does not aim to introduce fundamentally novel conclusions, it supports and strengthens the existing body of literature by providing additional real-world data. Further large-scale randomized controlled trials are warranted to confirm and expand upon these results.

This study has several limitations. First, it was conducted during the COVID-19 pandemic, which introduced challenges. Additionally, the cross-sectional design limits causal inference. The retrospective case–control design restricts conclusions about causality between MYO + ALA supplementation and the observed metabolic and hormonal changes. Despite conducting a power analysis with a sample size of 58 participants, the small sample size may limit generalizability and the ability to detect subtle effects, particularly in subgroup analyses.

The absence of a placebo control and blinding may introduce bias, and future studies with larger sample sizes and placebo-controlled designs are needed. Although Bonferroni correction was applied for multiple comparisons, the risk of Type I error remains. Furthermore, the lack of significance in some outcomes, such as HOMA-IR and LH/FSH ratio, may be attributed to the small sample size and short intervention duration. Larger studies with longer follow-up periods are needed to confirm these findings.

Finally, while the Ashwell classification and waist/height ratio have been proposed for metabolic risk assessment, their relevance and accuracy in PCOS patients require further validation.

## 5. Conclusions

The supplementation of ALA and MYO was found to assist in the maintenance of body weight and BMI in individuals with PCOS, whereas those who did not receive these supplements were more likely to experience an increase in body weight. Additionally, the mean HbA1c, insulin, and HOMA-IR values were comparable between the two groups, and the LH/FSH ratio significantly increased in the control group. LDL levels decreased significantly in the case group. MYO + ALA supplementation may contribute to metabolic and hormonal improvements in women with PCOS. Nonetheless, due to the retrospective nature of this study, these associations should be interpreted cautiously, and further large-scale randomized controlled trials are warranted. Further long-term studies with a large sample size are needed to evaluate the effects of MYO + ALA on reproductive physiology, hormonal and metabolic parameters, as well as to investigate the long-term effects and safety of MYO + ALA in patients with PCOS, and to further explore the mechanisms of action.

## Figures and Tables

**Table 1:** Anthropometric measurements in patients treated with ALA plus MYO and in the control group.

	Case Group (*n* = 29)			Control Group (*n* = 29)			*p* *#
	$\bar{X}\pm S.D.$			$\bar{X}\pm S.D.$			
Age	29.65 ± 4.20			29.31 ± 4.78			*p* = 0.772
	**Baseline**	**3 months**	*p* ^1^*#	**Baseline**	**3 months**	*p* ^2^*#	*p* ^3^**#
	$\bar{X}\pm S.D.$	$\bar{X}\pm S.D.$		$\bar{X}\pm S.D.$	$\bar{X}\pm S.D.$		
Weight (kg)	69.14 ± 10.68	68.37 ± 11.24	*p* = 0.484	68.36 ± 14.22	70.31 ± 15.78	***p* = 0.029**	*p* = 0.592
BMI (kg/m^2^)	26.62 ± 4.38	26.2 ± 4.45	*p* = 0.405	25.50 ± 4.63	26.20 ± 5.04	***p* = 0.026**	*p* = 0.998
Fat Percent (%)	32.40 ± 6.48	31.77 ± 6.58	*p* = 0.406	30.74 ± 6.86	31.78 ± 7.46	***p* = 0.026**	*p* = 0.998
BMR (kcal)	1475.98 ± 112.14	1468.47 ± 115.87	*p* = 0.475	1473.04 ± 139.67	1492.53 ± 152.46	***p* = 0.024**	*p* = 0.501

BMI: body mass index. BMR: basal metabolic rate. ^1,2,^* Repeated measurements were compared within the case–control groups. ^3,^** Repeated measurements of the case–control groups were compared. # Paired *t* Test.

**Table 2:** Comparison of biochemical measurements according to some anthropometric measurement classifications on a group basis for patients.

Case Group (*n* = 29)				Control Group (*n* = 29)		
$\bar{X}\pm S.D.$		$\bar{X}\pm S.D.$		$\bar{X}\pm S.D.$	$\bar{X}\pm S.D.$	
Variable	BMI < 25 kg/m^2^ (*n* = 11)	BMI ≥ 25 kg/m^2^ (*n* = 18)	*p* #	BMI < 25 kg/m^2^ (*n* = 11)	BMI ≥ 25 kg/m^2^ (*n* = 18)	*p* #
HbA1c (%)	5.51 ± 0.17	5.76 ± 0.32	***p*** ** = 0.013**	5.43 ± 0.21	5.86 ± 0.39	*p* = 0.172
HOMA-IR	2.82 ± 1.56	3.21 ± 1.61	*p* * = 0.528	2.25 ± 1.01	3.37 ± 2.31	*p* = 0.400
TG/HDL	2.05 ± 0.56	2.64 ± 1.48	*p* * = 0.719	1.39 ± 0.82	3.59 ± 2.44	***p*** * ** = 0.002**
	WC < 80 cm (*n* = 13)	WC ≥ 80 cm (*n* = 16)		WC < 80 cm (*n* = 14)	WC ≥ 80 cm (*n* = 15)	
HbA1c (%)	5.49 ± 0.16	5.80 ± 0.31	***p*** ** = 0.002**	5.44 ± 0.23	5.91 ± 0.38	***p*** ** < 0.001**
HOMA-IR	2.62 ± 1.51	3.43 ± 1.57	*p* = 0.168	2.22 ± 0.99	3.54 ± 2.39	*p* * = 0.239
TG/HDL	1.94 ± 0.59	2.81 ± 1.49	***p*** ** = 0.044**	1.39 ± 0.77	3.88 ± 2.45	***p*** * ** < 0.001**
	Waist/Hip < 0.85 (*n* = 24)	Waist/Hip ≥ 0.85 (*n* = 5)		Waist/Hip < 0.85 (*n* = 25)	Waist/Hip ≥ 0.85 (*n* = 4)	
HbA1c (%)	5.61 ± 0.28	5.92 ± 0.24	***p*** * ** = 0.020**	5.64 ± 0.39	5.95 ± 0.33	*p* = 0.149
HOMA-IR	3.25 ± 1.49	2.18 ± 1.81	*p* = 0.172	2.65 ± 1.79	4.51 ± 2.51	*p* * = 0.067
TG/HDL	2.29 ± 1.18	3.08 ± 1.44	*p* * = 0.326	2.17 ± 1.63	5.88 ± 2.95	***p*** * ** = 0.010**
	Waist/Height < 0.50 (*n* = 14)	Waist/Height ≥ 0.50 (*n* = 15)		Waist/Height < 0.50 (*n* = 13)	Waist/Height ≥ 0.50 (*n* = 16)	
HbA1c (%)	5.49 ± 0.15	5.82 ± 0.31	***p*** ** = 0.002**	5.42 ± 0.22	5.91 ± 0.36	***p*** ** < 0.001**
HOMA-IR	2.83 ± 1.66	3.28 ± 1.50	*p* = 0.452	2.30 ± 0.98	3.39 ± 2.39	*p* * = 0.511
TG/HDL	1.92 ± 0.56	2.89 ± 1.51	*p* = 0.093	1.37 ± 0.79	3.72 ± 2.43	***p*** * ** < 0.001**

BMI: body mass index. HOMA-IR: Homeostasis Model Assessment–Insulin Resistance. TG/HDL: triglyceride to high-density lipoprotein ratio. WC: waist circumference. # Independent sample *t* test; * Mann–Whitney.

**Table 3:** Biochemical profiles in patients treated with ALA plus MYO and in the control group.

Variable	Case Group (*n* = 29)		*p* ^1^*#	Control Group (*n* = 29)		*p* ^2^*#	*p* ^3^**#
	Baseline	3 Months		Baseline	3 Months		
	$\bar{X}\pm S.D.$	$\bar{X}\pm S.D.$		$\bar{X}\pm S.D.$	$\bar{X}\pm S.D.$		
Glucose (mg/dL)	95.10 ± 10.10	93.44 ± 1.06	*p* ^β^ = 0.437	95.72 ± 10.74	91.41 ± 10.47	*p* = 0.045	*p* = 0.477
HbA1c (%)	5.66 ± 0.42	5.67 ± 0.29	*p* = 0.892	5.66 ± 0.40	5.69 ± 0.39	*p* = 0.656	*p* ^β^ = 0.950
Insulin (μU/mL)	13.57 ± 8.56	13.24 ± 6.97	*p* = 0.885	10.47 ± 5.36	12.81 ± 8.31	*p* = 0.136	*p* = 0.570
HOMA-IR	3.28 ± 2.42	3.06 ± 1.57	*p* = 0.854	2.48 ± 1.29	2.91 ± 1.94	*p* = 0.265	*p* = 0.441
HDL (mg/dL)	49.91 ± 6.29	53.05 ± 9.00	*p* = 0.071	52.95 ± 11.89	52.96 ± 15.09	*p* = 0.957	*p* = 0.624
LDL (mg/dL)	128.02 ± 22.22	120.08 ± 25.16	***p*** ** = 0.024**	126.65 ± 30.32	119.14 ± 29.78	*p* = 0.090	*p* ^β^ = 0.87
Cholesterol (mg/dL)	190.85 ± 26.27	185.45 ± 30.51	*p* = 0.191	191.65 ± 34.01	186.31 ± 33.24	*p* = 0.294	*p* ^β^ = 0.630
TG (mg/dL)	119.97 ± 54.82	123.52 ± 60.19	*p* = 0.619	102.03 ± 49.51	120.14 ± 71.86	*p* = 0.116	*p* = 0.343
TG/HDL	2.45 ± 1.11	2.42 ± 1.23	*p* = 0.534	2.12 ± 1.34	2.68 ± 2.21	*p* = 0.127	*p* = 0.509
TSH (uIU/mL)	2.33 ± 1.10	2.90 ± 4.30	*p* = 0.370	1.89 ± 1.12	1.86 ± 1.14	*p* = 0.729	*p* = 0.152
Progesterone (ng/mL)	0.97 ± 2.40	0.59 ± 1.27	***p*** ** = 0.013**	0.65 ± 0.86	0.51 ± 1.34	***p*** ** = 0.006**	*p* = 0.276
E2 (pg/mL)	42.81 ± 16.78	57.57 ± 47.72	*p* = 0.165	62.81 ± 98.72	50.57 ± 59.05	*p* = 0.785	***p*** ** = 0.015**
LH (mlU/mL)	11.69 ± 8.68	11.18 ± 6.64	*p* = 0.561	10.39 ± 12.12	9.20 ± 5.69	*p* = 0.658	*p* = 0.111
FSH (mlU/mL)	6.23 ± 1.41	5.78 ± 1.40	*p* ^β^ = 0.190	7.59 ± 1.93	7.23 ± 4.21	***p*** ** = 0.005**	*p* = 0.078
LH/FSH	1.87 ± 0.35	1.49 ± 0.80	*p* = 0.325	1.34 ± 1.04	1.79 ± 0.92	***p*** ** = 0.010**	*p* = 0.199
CRP (mg/L)	1.45 ± 1.63	3.64 ± 6.58	***p*** ** = 0.041**	2.13 ± 2.48	3.21 ± 3.25	***p*** ** = 0.048**	*p* = 0.756

HbA1c: hemoglobin A1c. HOMA-IR: Homeostatic Model Assessment for Insulin Resistance. HDL: high-density lipoprotein. LDL: low-density lipoprotein. TG: triglycerides. TG/HDL: triglyceride to high-density lipoprotein ratio. TSH: thyroid-stimulating hormone. PRL: prolactin. DHEAS: dehydroepiandrosterone sulfate. tT: total testosterone. E2: estradiol. LH: luteinizing hormone. FSH: follicle-stimulating hormone. LH/FSH: luteinizing hormone to follicle-stimulating hormone ratio. CRP: C-reactive protein. ^1,2,^* Repeated measurements were compared within the case–control groups. ^3,^** Repeated measurements of the case–control groups were compared. ^β^ Paired *t* test; # Wilcoxon test.

**Table 4:** Examination of relationships between Ferriman–Gallwey score and subsequent biochemical measurements for groups and total.

Correlation	Ferriman−Gallwey Score					
	Case Group (*n* = 29)		Control Group (*n* = 29)		Total (*n* = 58)	
	*r*	*p* #	*r*	*p* #	*r*	*p* #
HbA1c (%)	0.193	0.316	−0.270	0.156	−0.039	0.772
Insulin (μU/mL)	−0.061	0.755	−0.334	0.077	−0.172	0.196
HOMA−IR	−0.056	0.775	−0.324	0.086	−0.152	0.256
DHEAS (ug/dl)	**0.388**	**0.037**	−0.135	0.486	0.208	0.117
Progesterone (ng/mL)	−0.080	0.678	0.042	0.829	0.018	0.891
E2 (pg/mL)	0.001	0.997	0.037	0.849	0.071	0.595
Androstenodione (ng/mL)	**0.388**	**0.038**	−0.074	0.702	**0.262**	**0.047**
LH (mlU/mL)	0.108	0.578	0.099	0.609	0.172	0.198
FSH (mlU/mL)	−0.010	0.961	0.035	0.859	−0.037	0.783
LH/FSH	−0.360	0.055	0.020	0.920	−0.226	0.087
CRP (mg/L)	−0.345	0.066	−0.123	0.524	−0.239	0.070

HbA1c: hemoglobin A1c. HOMA-IR: Homeostatic Model Assessment for Insulin Resistance. HDL: high-density lipoprotein. LDL: low-density lipoprotein. TG: triglycerides. TG/HDL: triglyceride to high-density lipoprotein ratio. TSH: thyroid-stimulating hormone. PRL: prolactin. DHEAS: dehydroepiandrosterone sulfate. tT: total testosterone. E2: estradiol. LH: luteinizing hormone. FSH: follicle-stimulating hormone. LH/FSH: luteinizing hormone to follicle-stimulating hormone ratio. CRP: C-reactive protein. # Spearman correlation.

## Data Availability

The data presented in this study are available on request from the corresponding author. The raw data supporting the conclusions of this article will be made available by the authors on request.
